# A hybrid organic–inorganic polariton LED

**DOI:** 10.1038/s41377-019-0180-8

**Published:** 2019-09-04

**Authors:** Rahul Jayaprakash, Kyriacos Georgiou, Harriet Coulthard, Alexis Askitopoulos, Sai K. Rajendran, David M. Coles, Andrew J. Musser, Jenny Clark, Ifor D. W. Samuel, Graham A. Turnbull, Pavlos G. Lagoudakis, David G. Lidzey

**Affiliations:** 1 Department of Physics and Astronomy, The University of Sheffield, Hicks Building, Hounsfield Road, Sheffield, S3 7RH UK; 2 Department of Physics and Astronomy, University of Southampton, Southampton, SO17 1BJ UK; 3 Skolkovo Institute of Science and Technology, Moskva, Russia; 4 Organic Semiconductor Centre, SUPA, School of Physics and Astronomy, University of St Andrews, St. Andrews, Fife, KY16 9SS UK; 5 Present Address: Baker Laboratory, Cornell University, 259 East Avenue, Ithaca, NY 14850 USA

**Keywords:** Polaritons, Organic LEDs, Inorganic LEDs, Polaritons, Organic LEDs

## Abstract

Polaritons are quasi-particles composed of a superposition of excitons and photons that can be created within a strongly coupled optical microcavity. Here, we describe a structure in which a strongly coupled microcavity containing an organic semiconductor is coupled to a second microcavity containing a series of weakly coupled inorganic quantum wells. We show that optical hybridisation occurs between the optical modes of the two cavities, creating a delocalised polaritonic state. By electrically injecting electron–hole pairs into the inorganic quantum-well system, we are able to transfer energy between the cavities and populate organic-exciton polaritons. Our approach represents a new strategy to create highly efficient devices for emerging ‘polaritonic’ technologies.

## Microcavities: Hybrid semiconductors team up for a quantum LED

Researchers have optically connected organic semiconductors and inorganic thin films to produce a light-emitting diode powered by quasi-particles that are part light and part matter. Polaritons, which form when electron–hole pairs in a semiconductor interact with photons, can be energy efficient sources of coherent light. Rahul Jayaprakash from the University of Sheffield in the United Kingdom and co-workers have now developed a device that generates electron-hole pairs inside a reflective microcavity filled with gallium–indium–phosphorus thin films. Then, they coupled the chamber’s optical resonance to a second microcavity containing light-absorbing phthalocyanine dye molecules. Time-resolved spectroscopy revealed that energy transfer from the inorganic to the organic microcavity creates sufficient polaritons densities for a visible-light LED, thanks to a delocalized quantum state that forms over both chambers.

## Introduction

Microcavity exciton-polaritons are hybrid light-matter quasi-particles formed from the coherent coupling of cavity photons and excitons^1^. Such phenomena occur in the strong coupling regime if the rate at which excitons and photons exchange energy is faster than their respective damping rates. As a result of their bosonic nature, cavity polaritons can undergo a range of many-body macroscopic quantum phenomena, including polariton lasing^2–5^, Bose–Einstein condensation^6^, superfluidity^7^ and vortex formation^8,9^.

The majority of research performed on strongly coupled microcavities has concentrated on structures containing a single type of semiconductor, either inorganic (e.g. GaAs^1^, ZnO^10^ and InGaN^11^) or organic (J-aggregates^12^, polymers^13^ and small molecules^14,15^). However, there is growing interest in the development of so-called ‘hybrid-semiconductor’ structures that contain both organic and inorganic materials. Here, optical hybridisation can occur between the various states within a cavity, realising ‘hybrid-polaritons’ that are described as an admixture of the cavity photon and different excitons. Interest in such systems originated from the work by Agranovich et al.^16^, who argued that exciton hybridisation could be harnessed to bypass phonon-relaxation bottlenecks^17^. Subsequent work also indicated that hybrid polariton states are expected to have enhanced optical non-linearity as a result of their hybrid Wannier/Frenkel exciton character^18^. Following such predictions, practical demonstrations of exciton hybridisation were made by combining GaInP and porphyrins^19^, GaAs and J-aggregates^20^, 2D-perovskites and phthalocyanines^21^, NTCDA molecular dyes and ZnO^22^ and J-aggregates and WSe_2_^23^. Although the predictions of enhanced relaxation or optical non-linearity have yet to be realised, experimental and theoretical work has shown that hybridisation of excitons in a microcavity can be used to facilitate long-range energy transfer via mixed-polariton states^22,24–26^.

In this paper, we explore a new type of hybrid-semiconductor heterostructure to facilitate energy transfer between semiconductor materials. Our structure is based on two microcavities that are optically coupled, with the semiconductor materials of interest (GaInP quantum wells (QWs) and a phthalocyanine dye) being placed in the two different cavities. Instead of hybridising the excitonic modes, we strongly couple the optical modes of the two cavities, resulting in the creation of an organic exciton polariton mode that is delocalised throughout the whole heterostructure. By generating electron–hole pairs in the inorganic QWs that are weakly coupled, we are able to pump the delocalised polariton mode and generate an emission from the organic exciton polariton. This approach allows separate optimisation of the organic and inorganic cavities, with coupling between cavities being an efficient means to transfer energy between organic and inorganic states. We expect that the improved design of the inorganic cavity device will allow us to increase the density of electrically injected polariton states to a point where non-linear effects can be anticipated. Our coupled-cavity structures are also of potential interest as a device platform in which to realise in-plane polariton entanglement^27–29^.

## Results

**Fig. 1:**
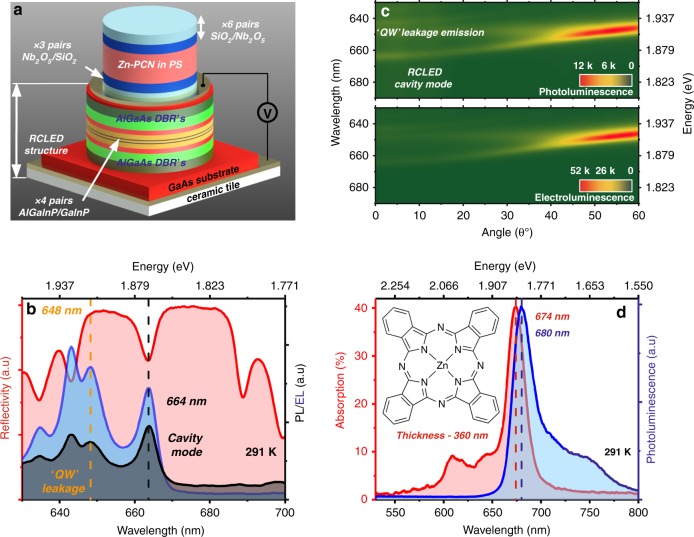
**a** A schematic of the structure of the coupled microcavity. Here, the top cavity contains a Zn-PCN/PS film positioned between two DBRs, and this structure is deposited onto an RCLED (bottom cavity) structure. **b** We plot the PL, EL and reflectivity of the RCLED at 291 K and normal incidence and indicate the peak of the QW emission and the location of the cavity mode. **c** Angle-dependent PL (top) and EL (bottom) from the RCLED structure. **d** We plot the absorption and PL from Zn-PCN/PS film on a fused silica substrate at 291 K. The chemical structure of the dye is shown in the inset

The structures we explored are based on a resonant cavity light-emitting diode (RCLED) onto which we fabricated a second cavity that contains an organic semiconductor, with the emission from the RCLED and the absorption/emission of the organic dye being almost degenerate in energy. Our approach is based upon previous work in which either organic or inorganic semiconductors placed in stacked cavities have been shown to undergo optical mixing and energy transfer^30–37^. The hybrid devices constructed are shown in Fig. 1a. Here, the inorganic component of the structure is based around a series of inorganic AlGaInP/GaInP QWs positioned between two Al_0.5_Ga_0.5_As/Al_0.92_Ga_0.08_As distributed Bragg reflectors (DBRs), with the DBRs being n- and p-type doped, such that this structure forms a RCLED. A second cavity was then fabricated onto the RCLED using a combination of electron-beam evaporation and spin-coating, with this ‘top cavity’ being either ‘empty’ (i.e., filled with a transparent SiO_2_ spacer layer) or containing the molecular dye zinc phthalocyanine (Zn-PCN) dispersed into a polystyrene (PS) matrix (see ‘Materials and methods’ for more details).

We characterised the optical properties of the organic and inorganic components of the heterostructure. Figure 1b plots the photoluminescence (PL) and electroluminescence (EL) emission emitted from the RCLED, along with its corresponding reflectivity. Here, the emission is at normal incidence and is concentrated around the RCLED cavity mode at 664 nm, although the QW emission is in fact centred at ~648 nm and has a linewidth of 28 meV at room temperature. This high negative detuning of the RCLED cavity mode with respect to the QW emission (~46 meV) results from the fact that we re-purposed RCLED structures that were originally designed as a light source for use in plastic optical fibre communication networks, with such devices emitting EL at a high off-axis angle. The angle-dependent PL (generated using light from a frequency-doubled Ti:sapphire laser at 445 nm) and EL from the RCLED structure are shown in Fig. 1c. The angular dispersion of PL and EL is identical, with most of the weakly coupled emission being concentrated around the spectral region 647–652 nm, corresponding to the peak emission intensity of the QWs.

Figure 1d plots the absorption, PL and chemical structure of Zn-PCN. This material has a relatively narrow 0–0 absorption transition at 674 nm (having a FWHM linewidth of 48 meV), together with weaker transitions at higher energies that result from vibronic coupling. The relatively high absorption coefficient of Zn-PCN together with its narrow linewidth and small Stokes shift (17 meV) confirm its suitability for strong coupling even at room temperature. We have specifically chosen Zn-PCN for this application because our hybrid device concept requires some degree of spectral overlap between the inorganic emission and the organic absorption. In the structures reported here, this entails the use of a dye absorbing in the wavelength range 665–680 nm, making Zn-PCN an ideal candidate. We note, however, that the same concept could easily be applied to other active-layer materials (e.g. tuneable hybrid perovskites^38^) or other organic–inorganic pairs operating in different spectral regions.

**Fig. 2:**
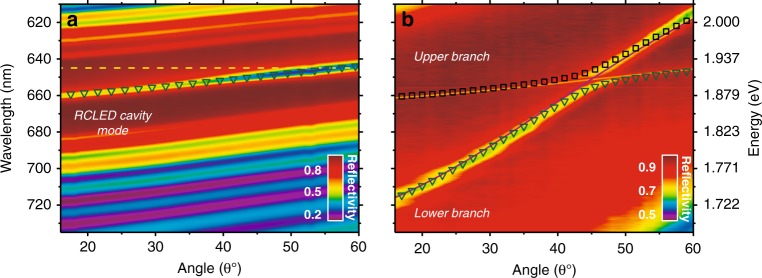
**a** The angle-dependent reflectivity from the RCLED structure. A 2 × 2 coupled oscillator model (symbols) has been used to simulate the cavity dispersion, where the QW emission (denoted by the dashed yellow line) is in the weak-coupling regime. **b** We show the angle-dependent reflectivity from an empty cavity deposited onto the RCLED structure. The dispersion of the lower and upper branches was again modelled using a 2 × 2 coupled oscillator model (symbols). The solid orange and purple lines correspond to the RCLED cavity mode and top cavity mode, respectively

We show below that energy transfer between the inorganic and organic components of Fig. 1a can occur via an optical mode that is delocalised through the cavity as a result of electromagnetic coupling between the two different cavities. To demonstrate this phenomenon, we first examined the optical properties of the inorganic cavity before deposition of the ‘top cavity’. This is shown in Fig. 1b, where we plot the white-light reflectivity of the AlGaInP/GaInP QW RCLED structure at normal incidence (see the schematic of this cavity in Supplementary Information Fig. S1). Here, it can be seen that the cavity is characterised by a DBR stopband in which a dip in reflectivity corresponds to a Fabry–Perot resonance. The linewidth of this optical mode is 22 meV, indicating a cavity Q-factor of 85. The angle-dependent reflectivity of this cavity is shown in Fig. 2a. As observed in the angular dispersion of EL and PL (see Fig. 1c), the cavity mode undergoes a blueshift as a function of increasing off-axis angle as expected^39^. We can describe this behaviour using a simple coupled oscillator model (as described in ‘Materials and methods’), in which the electronic transition of the AlGaInP/GaInP QW remains in the weak-coupling regime. We are confident of this assertion, as thermal energy at room temperature (~25 meV) greatly exceeds the GaInP QW exciton binding energy (8–12 meV^40^).

On top of the RCLED structure, a second optical cavity was fabricated as shown in Fig. 1a. We first discuss heterostructures in which the top cavity is ‘empty’ (i.e. simply containing an optically inert SiO_2_ spacer layer). The angle-dependent reflectivity of such a heterostructure is shown in Fig. 2b, and the PL emission from this empty cavity is presented in Fig. S2. Here, two optical modes can be detected that undergo anti-crossing at an angle of 45° with a splitting of ~27 meV between the modes. We propose that this anti-crossing results from strong coupling between the photon modes of the two cavities that are able to interact through the low-reflectivity central DBR. Here, we label the branches as the lower and upper branches and fit them using a two-level coupled oscillator model. We note that similar effects have been previously observed in inorganic coupled-cavity systems^34,36^, which are grown by MBE. However, as we show below, we can use our structures to also generate polaritonic states at room temperature following electrical injection into the weakly coupled RCLED.

**Fig. 3:**
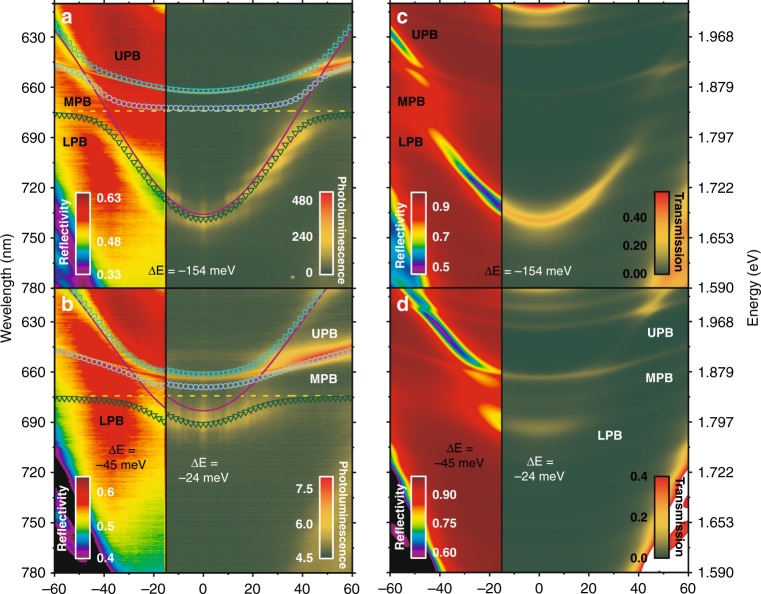
**a** Angle-dependent PL (right) and reflectivity (left) measurements from a cavity having a detuning of −154 meV. **b** Angle-dependent PL (right) and reflectivity (left) from a cavity having a detuning of −24 and −45 meV, respectively. In all cases, the dispersions of the LPB, MPB and UPB were calculated using a 3 × 3 coupled oscillator model (symbols). The solid orange and purple lines correspond to the RCLED cavity mode and the top cavity mode, respectively, and the dashed yellow line corresponds to the peak absorption of the Zn-PCN excitons. **c**, **d** Transfer matrix simulations of angle-dependent reflectivity and transmission that correspond to measured data shown in **a** and **b**

We now discuss the angle-dependent PL emission from heterostructures in which the top cavity contained the molecular dye Zn-PCN. Two cavities were studied in which the detuning of the top-cavity optical mode (relative to the energy of the Zn-PCN) was varied by adjusting the thickness of the Zn-PCN/PS layer. In the left-hand side of Fig. 3a, b, we plot the white-light optical reflectivity of such cavities, with the PL emission following non-resonant excitation at 445 nm (generated using a frequency-doubled Ti:sapphire laser) shown on the right. We first discuss the cavity dispersion extracted from the optical reflectivity measurements. In both cavities, we detect three optical branches, with these branches resulting from strong coupling between the Zn-PCN exciton and the two different cavity photon modes. We label the branches as the lower (LPB), middle (MPB) and upper (UPB) polariton branches and fit them to a three-level coupled oscillator model as described in ‘Materials and methods’. Our model indicates that the LPB and MPB undergo anti-crossing around the energy of the 0–0 Zn-PCN absorption, and the optical modes confined in the organic cavity and RCLED cavity undergo an anti-crossing and form the MPB and UPB. Specifically, the model predicts that the top-cavity mode in the structure shown in Fig. 3a has a negative detuning of −154 meV, and the LPB and MPB undergo a Rabi splitting of 52 meV. A Rabi splitting of 28 meV is also evident between the MPB and UPB. The cavity shown in Fig. 3b has a small negative detuning of −45 meV, with the Rabi splitting energy between the LPB and MPB being 52 meV.

**Fig. 4:**
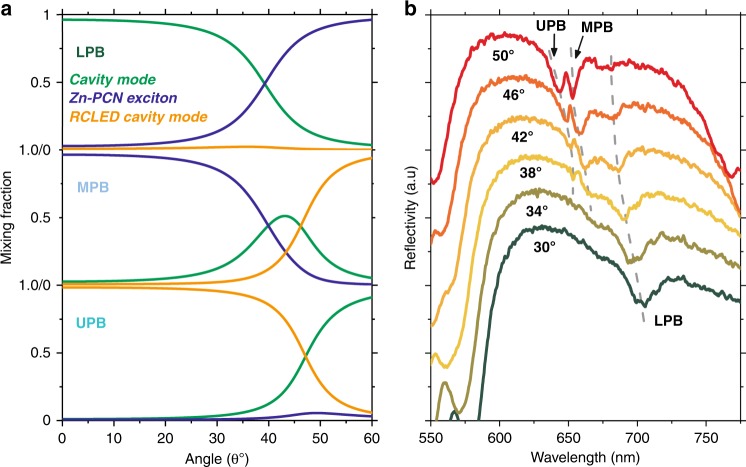
**a** The Hopfield coefficients for the LPB, MPB and UPB for a full device with a top cavity detuning of −154 meV. **b** Plots of reflectivity spectra recorded from a cavity having a detuning of −24 meV as a function of external angle

We can use our three-level coupled oscillator model to examine the mixing fractions of the different states in the three polariton branches (determined from optical reflectivity measurements), as shown in Fig. 4a. For the sake of simplicity, we present data for the cavity with a large negative detuning. Here, we find that the bottom of the LPB has a low Zn-PCN exciton fraction (3%), although the less negatively detuned cavity has a greater Zn-PCN fraction at the bottom of the LPB (17%). Importantly, we find that at an angle of 43°, the MPB is composed of equal amplitudes (24%) of Zn-PCN and the RCLED cavity mode. In Fig. 4b, we plot the reflectivity spectra from the same cavity recorded as a function of angle (line-cuts taken from the same data plotted as a colour map in Fig. 3a). Here, it can be seen that the MPB is clearly observed for angles in the range of 38–50°. The substantial mixing between Zn-PCN and the RCLED cavity mode suggests that radiative decay of electron–hole pairs within the GaInP QWs is likely to optically pump polariton states on the MPB. Such polaritons are expected to undergo efficient relaxation, creating excitons within the Zn-PCN reservoir^25^.

We now discuss the PL emission from our devices as shown in the right-hand side of Fig. 3a, b. It should be noted that the PL angular dispersion shown in Fig. 3b has been probed from a slightly different area of the sample from where reflectivity measurements were made, with the exciton-photon detuning being −24 meV. For both cavities, we find that the majority of the cavity emission is concentrated at ~648 nm, a wavelength that is coincident with the peak emission from the RCLED QWs. Significantly, we find that in both cavities, the LPB emits a small but significant fraction of luminescence. Here, we ascribe the relatively low emission intensity from the LPB to the low photoluminescence quantum yield (PLQY) of the Zn-PCN dye, which we have measured to be <1%. Note that in the cavity with a large negative detuning (Fig. 3a), a modulation can be seen in the intensity of the emission along the LPB; this effect occurs as a result of overlap between the (wide) DBR stopband of the organic cavity with the Bragg modes of the bottom inorganic cavity DBR that has a much narrower stopband. Likewise, the weak bands observed above the UPB in both reflectivity and emission simulations are related to the dispersion of the Bragg modes of the bottom inorganic cavity DBR.

Interestingly, the emission at ~648 nm apparently coincides with the dispersion of the uncoupled RCLED cavity mode rather than with that of the MPB or UPB. This behaviour is evident in both cavities explored. To understand such behaviour, we performed transfer matrix simulations (see ‘Materials and methods’) to model the angle-dependent optical reflectivity and transmission of the structures studied. The results of our modelling are shown in Fig. 3c, d for the structures whose measured reflectivity and PL emission are shown in Fig. 3a, b, respectively. There is a close correspondence between the measured and modelled angle-dependent reflectivity for both structures, indicating strong coupling and anti-crossing between the two different optical modes. Significantly, however, we find that modelled angular dependent transmission does not show clear splitting, as evidenced by the reflectivity measurements, and the linewidth of both branches is sufficiently broad that there is an optical pathway for a portion of the QW emission in the bottom cavity to leak out of the structure and coincide with the RCLED mode. We believe that the differences between the results of the reflectivity and transmission modelling most likely result from significant structural and compositional asymmetry between the top and bottom cavities^30^.

For completeness, we explored the optical properties of our cavities at low temperature, with our results presented in Fig. S3. Here, we find that although the GaInP QWs should contain excitons at 77 K, they still apparently operate in the weak-coupling regime. We believe that the absence of any apparent polariton splitting is a result of the relatively low Q-factor of the cavity (190). We also find that the emission from the organic cavity is essentially unchanged at low temperature; a result that is not particularly surprising as no fundamental change in the behaviour of Zn-PCN is expected over this temperature range.

**Fig. 5:**
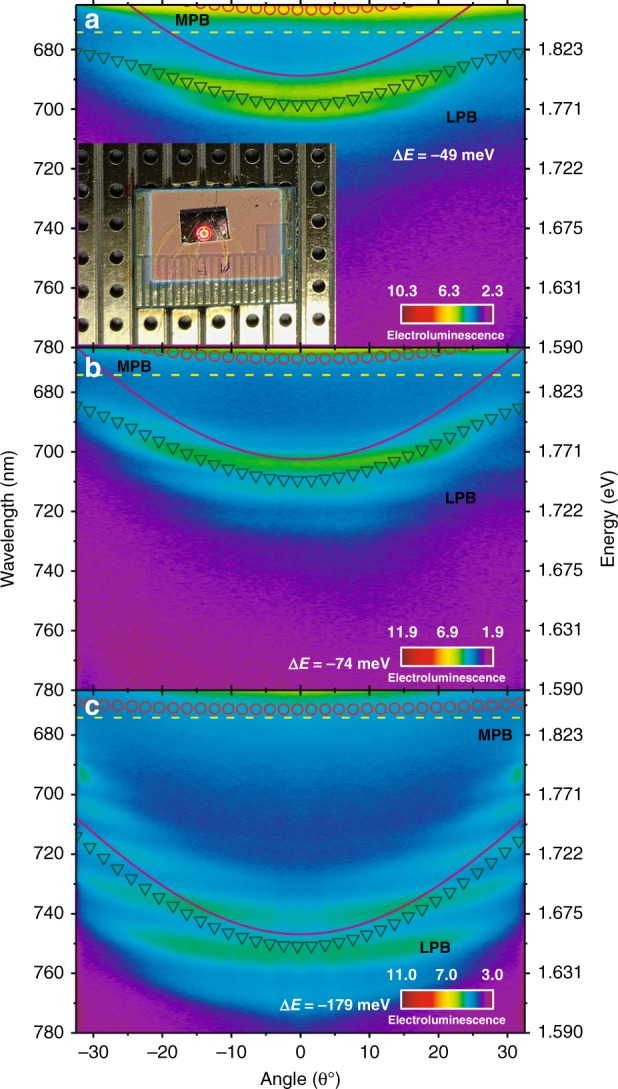
**a**–**c** EL emission (measured using a k-space imaging technique) from cavities having a detuning of −49, −74 and −179 meV, respectively. In all figures, the dispersion of the LPB was fitted using a 3 × 3 coupled oscillator model. The solid purple and dashed yellow lines correspond to the top cavity mode and the peak of the Zn-PCN exciton absorption, respectively. The inset in **a** is a photograph of the EL emission from a hybrid cavity

Our modelling of a mixed MPB suggests the possibility of energy transfer between the inorganic and organic systems. We propose that this mixed state is radiatively pumped^15,41,42^ at angles >45° by the weakly coupled GaInP QW emission, with which it is resonant. To probe this directly, we investigated the use of cavity structures similar to those reported in Fig. 3 to generate EL. All of these devices were also characterised with white-light reflectivity to confirm that they operated within the strong-coupling regime (see Supplementary Information Fig. S4). Devices were driven at room temperature with a voltage of 2–2. 1 V, a current density of ~2.7 kA/m^2^ and a total external quantum efficiency of 4.5% (although we estimate that only 2.5% of this emission originates from the LPB—an effect again ascribed to the low PLQY of the Zn-PCN dye). We also plotted the representative I–V curve shown in Supplementary Information Fig. S5. The angle-dependent EL from three different cavities with top cavity detuning of −49, −74 and −179 meV is presented in Fig. 5. We also include the modelled dispersion of the cavity mode (solid line) together with the LPB and MPB (symbols, Rabi splitting of 72 meV).

In all cases, we observe EL from the LPB with an energetic dispersion very similar to that of PL reported in Fig. 3, including similar intensity modulation along the LPB due to the Bragg modes of the RCLED mirror. We can thus be confident that the emission arises from the same states following electrical injection and photoexcitation. Importantly, the structure of our microcavity device ensures that electrical injection only occurs within the inorganic RCLED layer. Unlike under photoexcitation (Fig. 3), there can be no direct excitation of the organic system in this experiment. The observation of LPB emission thus requires energy transfer between the inorganic and organic materials. This transfer is accomplished over a remarkably long distance: the intervening DBR layers are ~1.4 µm thick, and we can thus rule out standard (dipole–dipole) energy transfer pathways that occur over a distance typically on the order of 10 nm. This distance also greatly exceeds that of the polariton-mediated energy transfer reported for pairs of molecular dyes contained within the same microcavity and coupled to the same photonic mode^24,43^. Instead, we propose that this transfer is mediated by the MPB, which mixes states in the top and bottom cavities and roughly coincides with the emission from the RCLED. This channel provides an efficient route to funnel energy into the LPB.

**Fig. 6:**
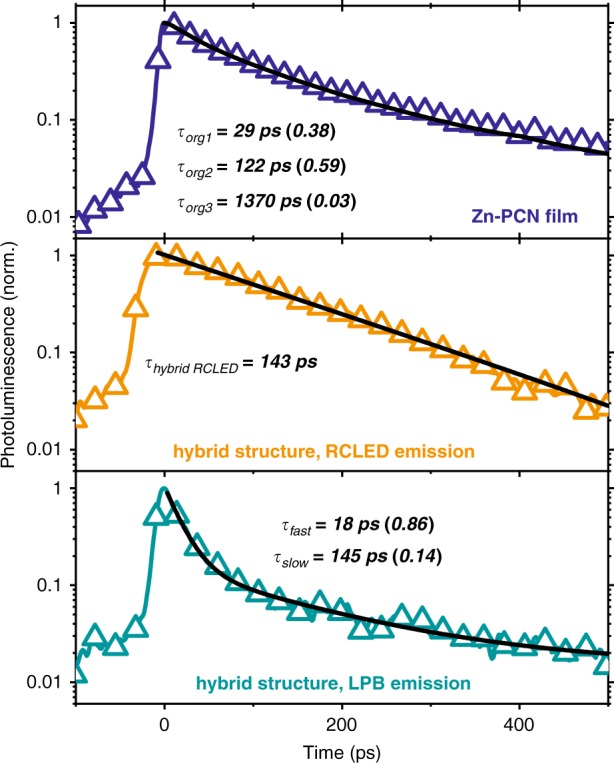
PL decay kinetics (symbols) for (top) a Zn-PCN/PS reference film detected at ~680 nm, (middle) the RCLED emission at ~657 nm measured from a hybrid cavity, and (bottom) emission from the LPB in the hybrid cavity (here measured at ~738 nm). The black solid lines are an exponential fit with the parameters shown

The EL measurements provide little detail on the underlying processes, so we performed PL lifetime measurements on full devices (see Fig. 6). In all cases, excitation was at 445 nm using 200 fs laser pulses, with the emission collected by an objective and detected using a streak camera. As a result of the large numerical aperture (NA) of the collection lens (NA = 0.55), the emission was collected from a range of angles estimated to be ±33.4°. Under these conditions, we directly excited states in both the inorganic and organic semiconductors. However, the similarity of the steady-state PL (Fig. 3) and EL (Fig. 5) suggests that the relaxation pathways in both cases are similar. In the top panel of Fig. 6, we plot the decay from a control Zn-PCN/PS film. The middle panel reports the decay of the direct RCLED emission at 657 nm within a hybrid structure with a large negative detuning (−154 meV), while the bottom panel shows the decay of the LPB emission (~738 nm) from the same structure.

We find that the Zn-PCN film undergoes complex multi-exponential decay, which we can fit to an equation with the form $I(t) = I_0 + ae^{ - \frac{t}{{\tau _0}}} + be^{ - \frac{t}{{\tau _1}}}$ + ⋯ where *I*(*t*) is the intensity at time *t*, *I*_0_ is the background intensity, and *a* and *b* are the amplitudes of the different decay terms with lifetimes *τ*_*i*_. Here, our best fit to the Zn-PCN decay dynamics reveals two dominant components with lifetimes of *τ*_org1_ = 29 ps and *τ*_org2_ = 122 ps and relative amplitudes of 0.38 and 0.59, respectively. These fast dynamics are likely related to energy migration and intermolecular interactions/aggregation within the film^44^ and are consistent with previous measurements of Zn-PCN^45,46^. There is an additional decay component with a small amplitude (0.03) and a long lifetime (1370 ps) that approaches the intrinsic lifetime of isolated Zn-PCN molecules^45,46^. In contrast, the decay of the RCLED emission is largely mono-exponential with a lifetime of 143 ps. Unsurprisingly, the decay of the LPB emission is also characterised by a number of transients. The fast component (18 ps) most closely agrees with the Zn-PCN exciton dynamics and is presumably related to direct excitation of the organic material, while the second component (145 ps) matches the decay of the RCLED emission. All of these lifetimes are much longer than the estimated polariton lifetime given by the cavity Q-factor (~22 fs), demonstrating that the emission is primarily mediated by longer-lived states in the (organic) exciton reservoir. The observation that the LPB emission tracks the lifetimes of both organic and inorganic components suggests that the same energy transfer process observed in EL is at play here. Note that we have also studied the PL emission lifetime from other cavities having different energetic detuning and find in all cases that the dynamics of the RCLED and LPB emissions are very similar (see Supplementary Fig. S6).

Combining the steady-state and time-resolved measurements in Figs. 3, 5 and 6, we built the following picture to describe our results. Photoexcitation generates electron–hole pairs in the GaInP QWs and Zn-PCN excitons in the top cavity. The Zn-PCN excitons are able to populate states on the LPB, most likely by either a vibrational scattering mechanism^47^ or by direct radiative pumping^15,42^. Excitations in GaInP will behave similarly regardless of how they are generated. The longer component of the LPB decay and the EL together indicate that the LPB is also indirectly populated by the slower, radiative decay of electron–hole pairs in the GaInP QWs. Our analysis of the angle-resolved data in Fig. 3 suggests that such a population process most likely proceeds via direct radiative pumping of the MPB states, which undergo rapid relaxation to the Zn-PCN reservoir and then populate the LPB.

This model shows that our devices undergo inorganic–organic energy transfer, although this occurs without direct hybridisation of the electronic states, as proposed by Agranovich et al. (here, the inorganic QWs remain weakly coupled)^16,18^. Instead, the approach is based on coupling two systems that are separately tuneable and can potentially offer enhanced electrical performance over devices in which both organic and inorganic components are located within the same cavity.

To explore the extent to which our RCLED devices can act as a light source to generate a large population of polaritons, we investigated their ability to generate EL at a high drive bias by applying 250 ns voltage pulses of 70 V at 20 kHz (corresponding to a continuous wave [CW] current of ~13 kA/m^2^). This generated an optical power per pulse from the device of ~50 nJ/cm^2^. Clearly, this pump power is significantly below that required to reach the threshold in an organic polariton laser, which is typically 500 μJ/cm^2^ ^48^. We also characterised the relative intensity of the RCLED and LPB emissions as a function of the CW injection current density and found that no non-linearities are observed at current densities of 70 kA/m^2^ (see Supplementary Fig. S8), suggesting that significant further optimisation of the inorganic microcavity device will be required to approach condensation thresholds.

We also made preliminary measurements of the stability of the k-space EL emission over a period of 35 h (see Supplementary Fig. S7) and recorded the integrated intensity of the RCLED and LPB emission intensity as a function of time on the same sample discussed in Fig. 5b. Here, the intensity of the RCLED leakage emission remained essentially constant over this period, however the LPB underwent a reduction in emission intensity by ~50%. We believe that this drop in the LPB emission intensity is most likely due to a combination of gradual photooxidation of Zn-PCN and possible thermal effects that may slowly anneal the organic layer, changing its morphology and the density of the quenching sites. We emphasise, however, that our device structures are not currently optimised to maximise long-term stability, and no active steps have been taken to reduce the ingress of oxygen into the device. Significantly, however, such structures are fundamentally different from that of an organic LED, as all charge injection and recombination occurs within the photochemically robust inorganic semiconductor. Thus, our devices circumvent the most detrimental process in organic LEDs—charge recombination in the organic layer, (which forms non-emissive and reactive triplet excitons)—and with suitable optimisation, they are likely to have longer lifetimes as a result.

## Discussion

The structures we have fabricated offer possible opportunities to create new types of laser devices. Furthermore, the mixing between optical modes in coupled cavities presents a new opportunity to generate polarisation-entangled polaritons through parametric scattering as described by Einkemmer et al.^29^. In this process, polariton pairs from the MPB at *k* = 0 would scatter to the LPB at *k* = *q*, *k*′ = −*q* while preserving the energy, momentum and spin of the initial states. The generated polariton pairs thus propagate in opposite in-plane directions and have a spin degree of freedom that is entangled and is both dependent on the scattering process and spin configuration of the initial polariton pair. More complex schemes for the generation of hyperentangled (entangled also in energy) polariton pairs have also been proposed^28^.

In summary, we have fabricated a series of structures based on two coupled microcavities containing a strong-coupled organic molecular dye Zn-PCN and a series of weakly coupled GaInP QWs. We demonstrate optical coupling between the two cavities via linear spectroscopy and optical modelling. Time-resolved spectroscopy is used to demonstrate that polariton states on the LPB undergo optical decay with a transient that shows characteristics of both the organic exciton and the weakly coupled GaInP QWs. Our measurements indicate a mechanism in which emission from the weakly coupled GaInP QWs is able to optically pump states on the MPB that are delocalised throughout the two coupled cavities. The rapid decay of the MPB states results in the generation of excitons in the Zn-PCN reservoir that then populate the states on the LPB. We utilise this concept directly in a practical device, achieving visible-light emission from a hybrid polariton LED operating at room temperature. Our approach has the advantage that both organic and inorganic cavities can be separately optimised, presenting a route to structures in which a high density of polariton states can potentially be generated via electrical injection. Such structures that rely on coupling and hybridisation between two independent cavity modes offer opportunities to develop devices that will be of interest in the emergent field of quantum polaritonics.

## Materials and methods

### Preparation of organic semiconductor

A 1 mL solution containing 50 mg/mL PS in toluene was prepared, to which 2 mg of Zn-PCN was added. The solution was stirred for 90 min at 75 °C and then filtered using a PTFE filter. This solution was then spin-coated onto a fused silica substrate at 2000 rpm, creating an ~360-nm-thick film. This film was then used in experiments to measure PL, absorption and transient luminescence.

### Fabrication of DBR mirrors

DBRs were deposited via electron-beam (e-beam) evaporation in an Angstrom Engineering deposition chamber held at a base pressure of 2 × 10^−6^ mBar. The chamber contained two Telemark 25 cc e-beam sources equipped with high-strength graphite crucibles filled either with SiO_2_ or Nb_2_O_5_ (TiO_2_ in the electrical devices).

### Fabrication of hybrid monolithic structures

The RCLED wafer used is based on two AlGaAs DBRs placed on either side of four AlGaInP (barrier)/GaInP QWs. Here, the top and bottom mirrors are p- and n-doped, respectively, and form a λ-cavity. To fabricate the RCLED devices, the wafer supplied by Firecomms Ltd. was etched using reactive ion etching, creating closely spaced (~0.1 mm) circular mesas with a diameter of ~1 mm, with the etch depth extending into the bottom mirror. Gold ring contacts with an inner diameter of ~0.6 mm were then thermally evaporated on to the circular mesas in which the top layer was formed from heavily p-doped GaAs, which acted as a current spreading layer. A DBR consisting of three pairs of SiO_2_/TiO_2_ was then deposited onto the mesa, followed by the deposition of a 500-nm-thick layer of Zn–PCN/PS. The cavity was then completed by depositing an additional eight pairs of TiO_2_/SiO_2_ DBR by e-beam evaporation onto the surface of the Zn-PCN/PS. This structure was then mounted on to a ceramic tile, with the negative and positive contacts of the individual devices made through the substrate and gold ring contacts, respectively. The structures used for optical characterisation were very similar, although the pre-processing steps prior to DBR fabrication were not performed. Moreover, TiO_2_ was replaced by Nb_2_O_5_, and the final top DBR consisted of six rather than eight pairs. As a control, ‘empty cavity’ structures were also fabricated in which the active Zn-PCN/PS layer was replaced by a SiO_2_ spacer layer.

### Optical measurements

#### Basic optical characterisation

Angle-dependent PL, EL, reflectivity and absorption measurements were performed using an Andor Shamrock SR-303i-A triple-grating imaging spectrograph with a focal length of 0.303 m. The spectra were recorded using a 300 groove/mm grating blazed at 500 nm. For PL measurements, the samples were excited using a pulsed, frequency-doubled Ti:sapphire (coherent MIRA 900) laser at 445 nm, having a pulse width of ~150 fs and a nominal pulse repetition rate of ~76 MHz. The reflectivity and absorption measurements were performed using a fibre coupled 20 W tungsten halogen light source (Ocean Optics DH-2000-BAL).

#### Goniometer setup

Angle-dependent reflectivity measurements were made using a goniometer setup consisting of two motorised arms fixed to a common rotation stage, where one arm was used for excitation while the other for collection. White light from the fibre coupled source was focused on to the sample using a pair of lenses and collected using a second pair of lenses, which was then fibre coupled to the spectrometer. A 100-µm pinhole at the focus of the final collection lens spatially filtered the signal into the fibre that was coupled to the spectrometer. The setup allowed reflectivity measurements to be made over an angular range of 15°–60° in steps of 1°. A vertically displaced third arm was used for PL excitation, in which the laser was focussed on to the sample using a 100-mm focal-length lens at an angle of incidence of ~15°. This configuration allowed PL to be collected over an angular range of 0°–60°. For absorption measurements, the excitation arm was configured at normal incidence, with the white light transmitted through the sample collected using a pair of lenses on a fourth arm, which was then fibre coupled to the spectrometer. Low-temperature measurements were performed by replacing the sample holder on a three-axis stage with an Oxford Instruments D10200 bath cryostat.

#### K-space imaging

We used k-space imaging to measure EL and PL emission. Here, the laser was focused on the sample at normal incidence using an aspherical lens with a NA = 0.63, with the PL signal collected through the same optical path using a beam splitter. This light was then focussed into the spectrometer using a final collection lens. An additional Fourier-plane imaging lens positioned before the final lens facilitated the Fourier plane to be imaged by the spectrometer. Here, a pinhole was positioned at the focus of the imaging lens (before the final collection lens), which allowed the emission to be spatially filtered, permitting the unwanted real space signal to be rejected. For k-space imaging of EL emission, a DC bias was applied to the device using a Keithley 2602 sourcemeter.

#### PL characterisation of the organic film

PL measurements were performed using an Andor Shamrock 303i-B triple-grating imaging spectrograph with a focal length of 0.303 m. The spectra were acquired using a 150 grooves/mm grating blazed at 500 nm. A frequency-tripled Nd-YAG (Innolase) laser was used to excite the sample at 355 nm, having a pulse width of ~1 ns and a pulse repetition rate of ~5 kHz. The laser was focused on the sample at an angle of incidence of ~40°, through the edge of a high NA = 0.76 aspherical lens, with the optical axis aligned with the normal to the sample plane. The collimated PL from the sample was then focussed into the spectrometer using a final collection lens.

#### Normal incidence characterisation of the RCLED structure

The PL, EL and reflectivity measurements were performed using the goniometer setup. A frequency-doubled Ti:sapphire (coherent MIRA 900) laser at 445 nm with a pulse width of ~150 fs and a nominal pulse repetition rate of ~76 MHz was used for PL excitation. For EL measurements, a DC bias was applied to the devices using a Keithley 2602 sourcemeter. Reflectivity measurements were made by placing a beam splitter in the excitation arm (at normal incidence), with the light reflected from the sample being fibre coupled to the spectrometer using a final collection lens.

#### Time-resolved PL measurements

The ultrafast spectroscopy measurements were performed using a streak camera coupled to a 300-mm spectrometer equipped with a 1200 groove/mm grating. The non-resonant optical excitation at 445 nm was provided by a frequency-doubled Ti:sapphire oscillator with a pulse width of <200 fs and a repetition rate of 78.6 MHz. Our system has an energy resolution of 0.5 meV and a time resolution of 1.5 ps. Images acquired with this system were corrected for energy-time uncertainty effects (curvature correction), and the transient decays were extracted by integrating over the desired energy range corresponding approximately to the linewidth of the respective energy mode.

#### Time-resolved PL measurements on the organic film

The emission lifetime from the Zn-PCN films was measured using a Hamamatsu C10910 streak camera. The films at room temperature in a vacuum atmosphere were excited using 320 nm pulses of 200 fs duration at a 100 kHz repetition rate. The femtosecond pulses were generated from a Pharos femtosecond laser system from Light Conversion (SP-06-200-PP), and 320 nm wavelength pulses were generated using an Orpheus optical parametric amplifier. The emitted spectra were resolved using a Princeton Instruments Acton SpectraPro SP-2300 and a Hamamatsu M10911 synchroscan unit coupled to a C10600 camera.

#### PLQY measurements

The PLQY was measured using a Hamamatsu Photonics Absolute quantum yield spectrometer (C9920-02). The excitation wavelength from the xenon lamp was selected using the monochromator, and the emitted light from the film was collected using a PMA-12 (C10027-01) photonic multichannel analyser in an integrating sphere. The experiments were performed in an environment of air as well as nitrogen.

### Optical modelling

#### Coupled oscillator model

We describe the coupling among the RCLED cavity optical-mode (*E*_RCLED_), the cavity mode (*E*_cav_) and the 0–0 transition (*E*_org_) of the Zn-PCN using the following Hamiltonian

$$\left[ {\begin{array}{*{20}{c}} {E_{cav}\left( {k_{||}} \right) - i\gamma _{cav}} & {g_1} & {g_2} \\ {g_1} & {E_{org}\left( {k_{||}} \right) - i\gamma _{org}} & 0 \\ {g_2} & 0 & {E_{RCLED}\left( {k_{||}} \right) - i\gamma _{RCLED}} \end{array}} \right]$$

where *γ*_cav_, *γ*_org_ and *γ*_RCLED_ correspond to the half-width half maximum of the cavity mode, organic transition and RCLED cavity mode, respectively. *g*_1_ and *g*_2_ are the respective coupling strengths of the organic transition and the RCLED cavity mode with the top cavity mode.

#### Transfer matrix model

A transfer matrix model (which is described in detail in the *‘Basics of optics of multilayer systems’* ^49^) was used to calculate the angle-dependent reflectivity (*R*) and transmission (*T*) of the coupled-cavity structure. As input to the model, the absorption spectrum of the organic active layer was fitted using three Lorentzian functions, with the amplitude of the functions effectively corresponding to the excitonic oscillator strength.

### Electrical characterisation

#### Continuous voltage measurements

A dual channel Keithley 2602 sourcemeter was used to apply bias to the hybrid LEDs and simultaneously measure the current flowing through the device.

#### Pulsed voltage measurements

An Agilent 8114A pulse generator (100 V/2 A) with a frequency range of 1 Hz to 15 MHz and a pulse-width range of 10 ns to 150 ms was used to apply bias to the LEDs. A Keithley 2602 sourcemeter was then used to measure the average current flowing through the devices.

#### EL external quantum efficiency (EQE) measurements

Device EQE was estimated from the total luminance as described in ref. ^50^. The total luminance was measured using a Konica Minolta luminance metre equipped with an LS-110 zoom lens over an angular range of ±9°. The value of EQE reported includes emission from both the LPB and the RCLED.

## Supplementary information

Supplementary Information: A hybrid organic-inorganic polariton LED
