# Supplementary material for: A hybrid organic–inorganic polariton LED
Source: Light Sci Appl. 2019 Sep 4;8:81. doi: 10.1038/s41377-019-0180-8 (PMC6804868; doi:10.1038/s41377-019-0180-8)
Supplement: Supplementary file 1 — Supplementary Information: A hybrid organic-inorganic polariton LED [file 41377_2019_180_MOESM1_ESM.docx]

**A hybrid organic-inorganic polariton LED: Supplementary Information**

Rahul Jayaprakash^1^, Kyriacos Georgiou^1^, Harriet Coulthard^1^, Alexis Askitopoulos^2,3^, Sai K. Rajendran^4^, David M. Coles^1^, Andrew J. Musser^1,#^, Jenny Clark^1^, Ifor D. W. Samuel^4^, Graham A. Turnbull^4^, Pavlos G. Lagoudakis^2,3^ and David G. Lidzey^1,*^

^1^Department of Physics and Astronomy, The University of Sheffield, Hicks Building, Hounsfield Road, Sheffield S3 7RH, U.K.

^2^Department of Physics and Astronomy, University of Southampton,

Southampton, SO17 1BJ, U.K.

^3^Skolkovo Institute of Science and Technology, Russian Federation

^4^Organic Semiconductor Centre, SUPA, School of Physics & Astronomy, University of St Andrews, St. Andrews, Fife, KY16 9SS, U.K.

^#^Current address: Baker Laboratory, Cornell University, 259 East Avenue, Ithaca, New York 14850, United States

[*r.jayaprakash@sheffield.ac.uk*](mailto:r.jayaprakash@sheffield.ac.uk)*,* [*k.georgiou@sheffield.ac.uk*](mailto:k.georgiou@sheffield.ac.uk)*,* [*hcoulthard1@sheffield.ac.uk*](mailto:hcoulthard1@sheffield.ac.uk)*,* [*a.askitop@gmail.com*](mailto:a.askitop@gmail.com)*,* [*skr7@st-andrews.ac.uk*](mailto:skr7@st-andrews.ac.uk)*,* [*davcoles@googlemail.com*](mailto:davcoles@googlemail.com)*,* [*a.musser@sheffield.ac.uk*](mailto:a.musser@sheffield.ac.uk)*,* [*jenny.clark@sheffield.ac.uk*](mailto:jenny.clark@sheffield.ac.uk)*,* [*idws@st-andrews.ac.uk*](mailto:idws@st-andrews.ac.uk)*,* [*gat@st-andrews.ac.uk*](mailto:gat@st-andrews.ac.uk)*,* [*pavlos.lagoudakis@soton.ac.uk*](mailto:pavlos.lagoudakis@soton.ac.uk)*, Corresponding author:* *[*d.g.lidzey@sheffield.ac.uk*](mailto:d.g.lidzey@sheffield.ac.uk)

**Abstract**

Polaritons are quasi-particles composed of a superposition of excitons and photons that can be created within a strongly coupled optical microcavity. Here, we describe a structure in which a strongly coupled microcavity containing an organic semiconductor is coupled to a second microcavity containing a series of weakly coupled inorganic quantum wells. We show that optical hybridisation occurs between the optical modes of the two cavities, creating a delocalised polaritonic state. By electrically injecting electron-hole pairs into the inorganic quantum-well system, we are able to transfer energy between the cavities and populate organic-exciton polaritons. Our approach represents a new strategy to create highly efficient devices for emerging ‘polaritonic’ technologies.

**RCLED structure**

**
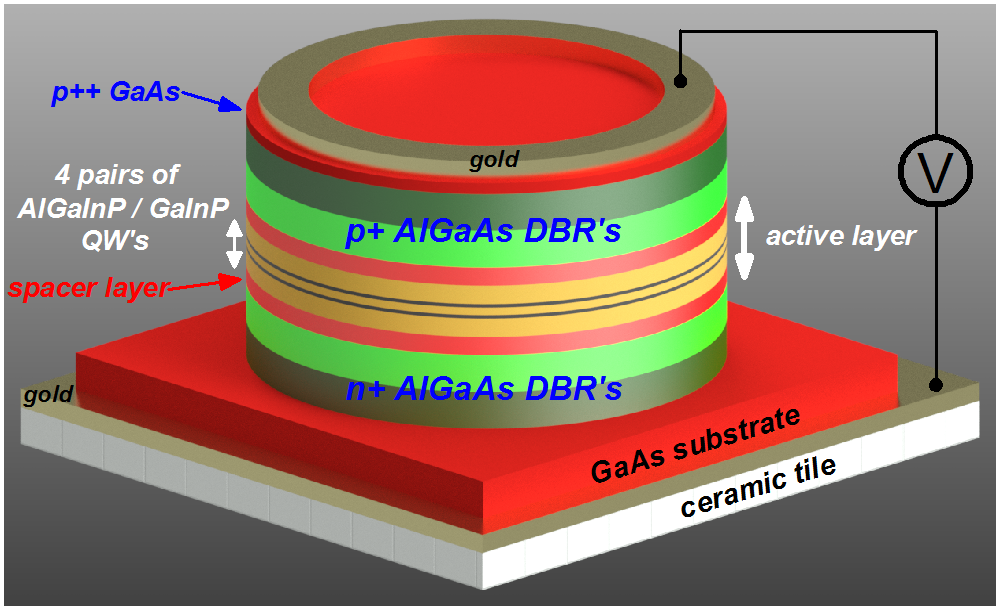
**

Figure S1: A schematic describing the structure of the RCLED device.

**Angle-dependent PL from the empty-cavity structure**


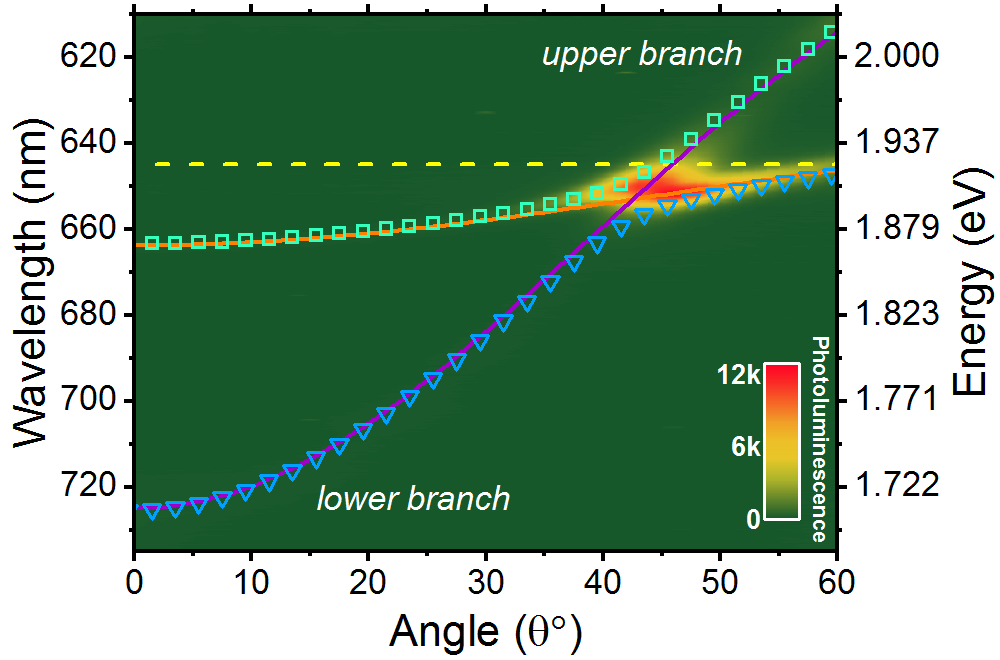


Figure S2: We plot the angle-dependent reflectivity from an empty cavity deposited onto the RCLED structure. The dispersion of the lower and upper branches has been modelled using a 2 x 2 coupled oscillator model (symbols). The solid orange and purple lines correspond to the RCLED cavity mode and top cavity mode respectively.

PL emission from an empty-cavity structure corresponding to the reflectivity discussed in Figure 2, is shown in Figure S2. Here, PL emission and reflectivity were measured from a slightly different area of the sample. Here, we overlay the results of a coupled oscillator model in which the QW excitons are weakly coupled (line and symbols). It can be seen that most of the emission coincides with the RCLED cavity mode; an observation in line with those made the hybrid cavities discussed in Figure 3 of the main paper. Our simulations show that the lower branch is degenerate in energy with the RCLED leakage above the anti-crossing point. We also observe some emission from the upper branch which was not observed in the hybrid cavities. We believe that the ‘darkness’ of the upper branch in the hybrid cavity results from rapid relaxation of polaritons to the organic reservoir.

**Temperature-dependent measurements on hybrid cavities**

**
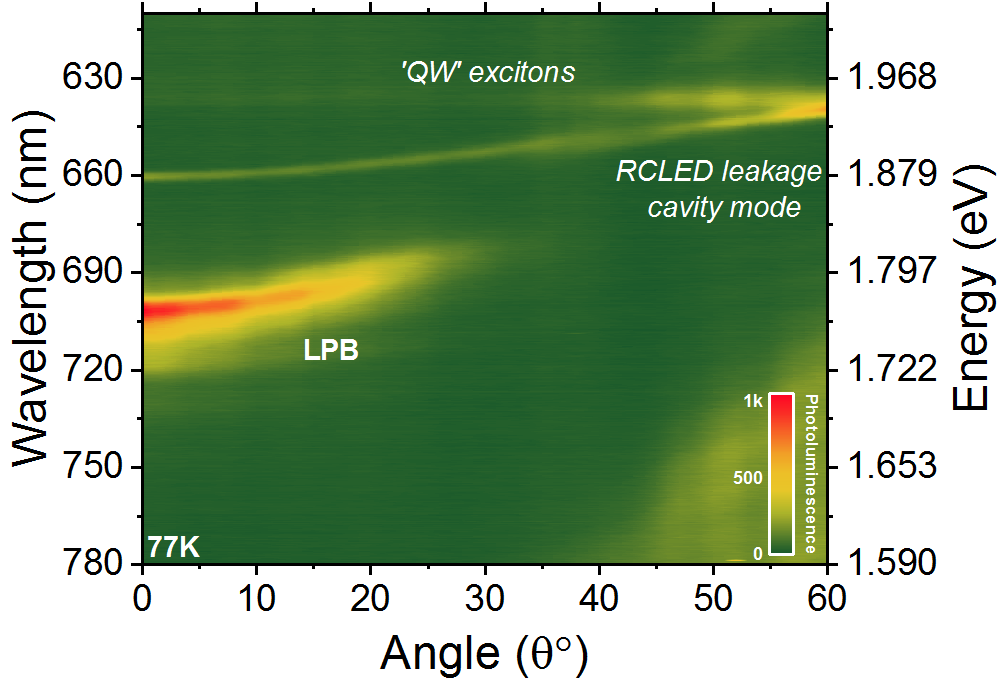
**

Figure S3: Angle-dependent PL measurements from a hybrid cavity at 77K.

The PL from the sample shown in Figure 3(a), is shown in Figure S3(a) at 77K. Here, emission is observed around 636.5 nm that does not have any angular dispersion, which we believe corresponds to emission from QW excitons. We note that emission from the RCLED cavity does not apparently cross the QW excitons suggesting that it remains in the weak coupling regime even at 77K. We believe that the QWs most likely remain in the weak-coupling regime as a result of the low Q-factor of the RCLED cavity mode 190 (k=0), and the fact that the reflectivity of the DBR is significantly reduced at high angle.

**Angle-dependent reflectivity from a hybrid polariton LED**


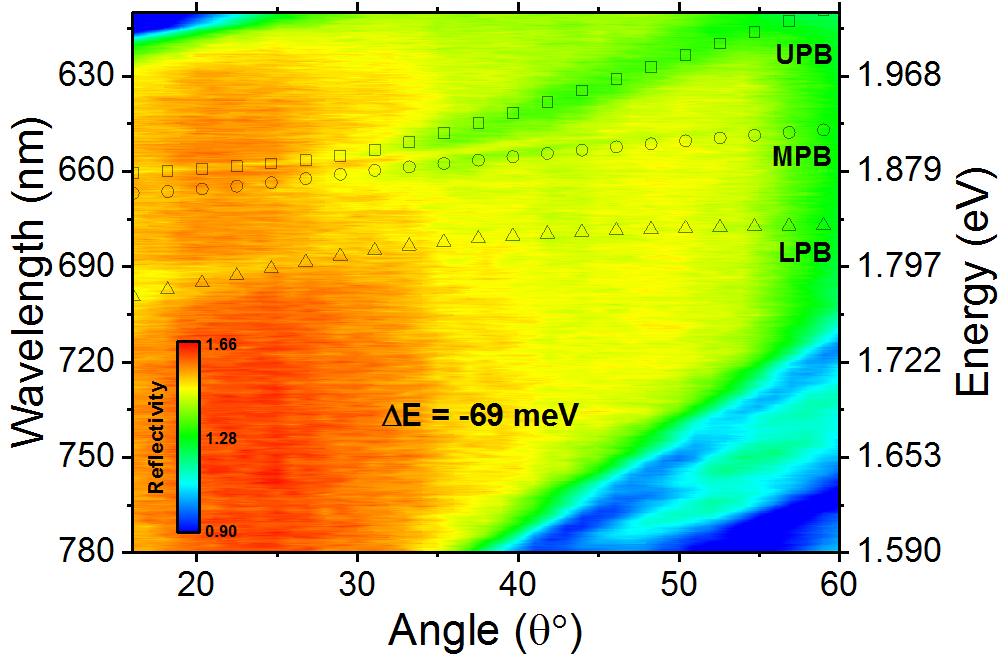


Figure S4: Angle-dependent reflectivity from a hybrid polariton LED, corresponding to a detuning of -69 meV. The dispersion of LPB, MPB and UPB have been derived from a 3 x 3 coupled oscillator model.

The angle- dependent reflectivity from a hybrid polariton LED, corresponding to a detuning of -69 meV is shown in Figure S4. Here, we include the results of a 3-level coupled oscillator model showing the dispersion of LPB, MPB and UPB, where the Rabi-splitting between the LPB and MPB is 72 meV.

**IV curve from a hybrid polariton LED**

**
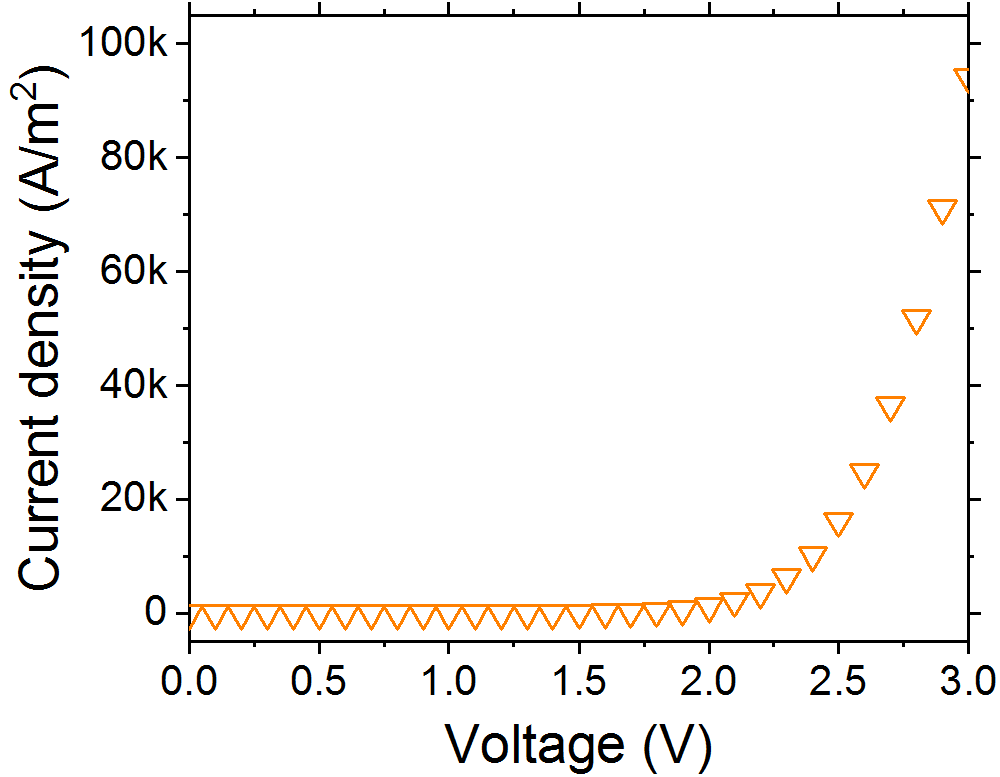
**

Figure S5: IV curve from a hybrid polariton LED

**LPB decay lifetime from a hybrid cavity corresponding to a detuning of -24 meV**

**
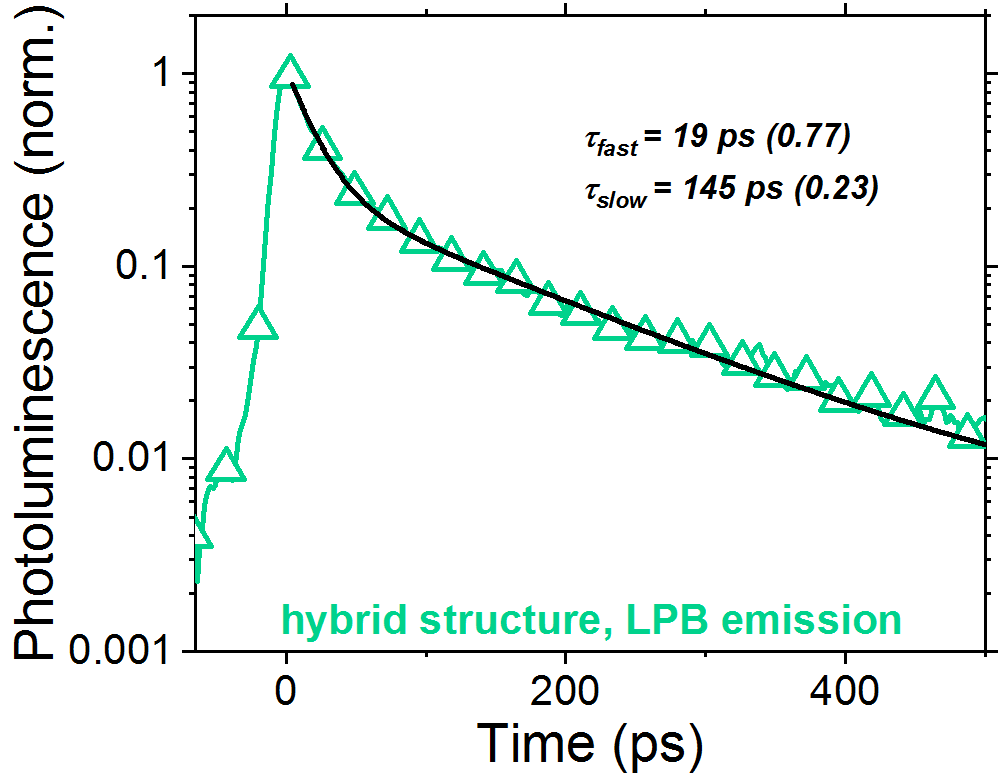
**

Figure S6: PL decay kinetics (symbols) for the LPB in the hybrid cavity measured at ~ 691 nm. The black solid line is an exponential fit with parameters shown.

**Stability measurements**

Stability measurements have been performed on the sample discussed in Figure 5(b), over a period of 35 hours. The integrated EL intensity corresponding to the LPB and RCLED

leakage emission extracted from the k-space image, is shown in Figure S7.

**
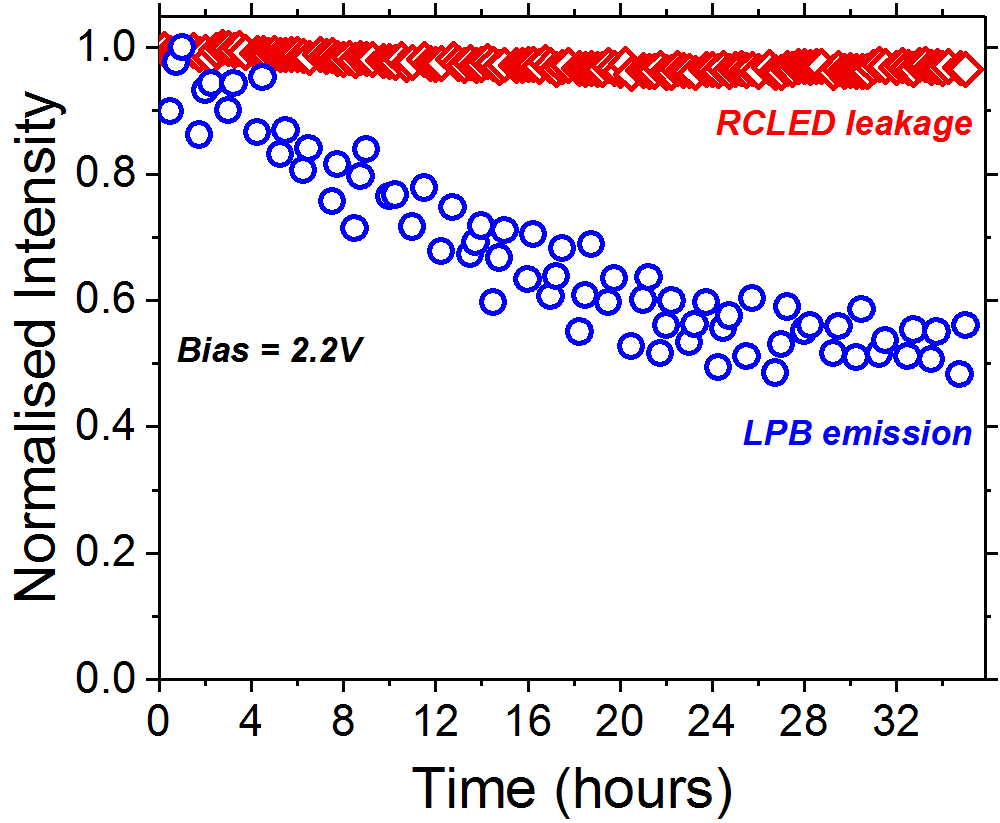
**

Figure S7: Integrated EL intensity over a period of 35 hours, where blue circles and red squares correspond to LPB and RCLED leakage emission respectively

**Integrated intensity as a function of LED current density**

The relative intensity of LPB and RCLED leakage emission as a function of current density was extracted from integrating the relevant features in k-space emission spectra over an

**
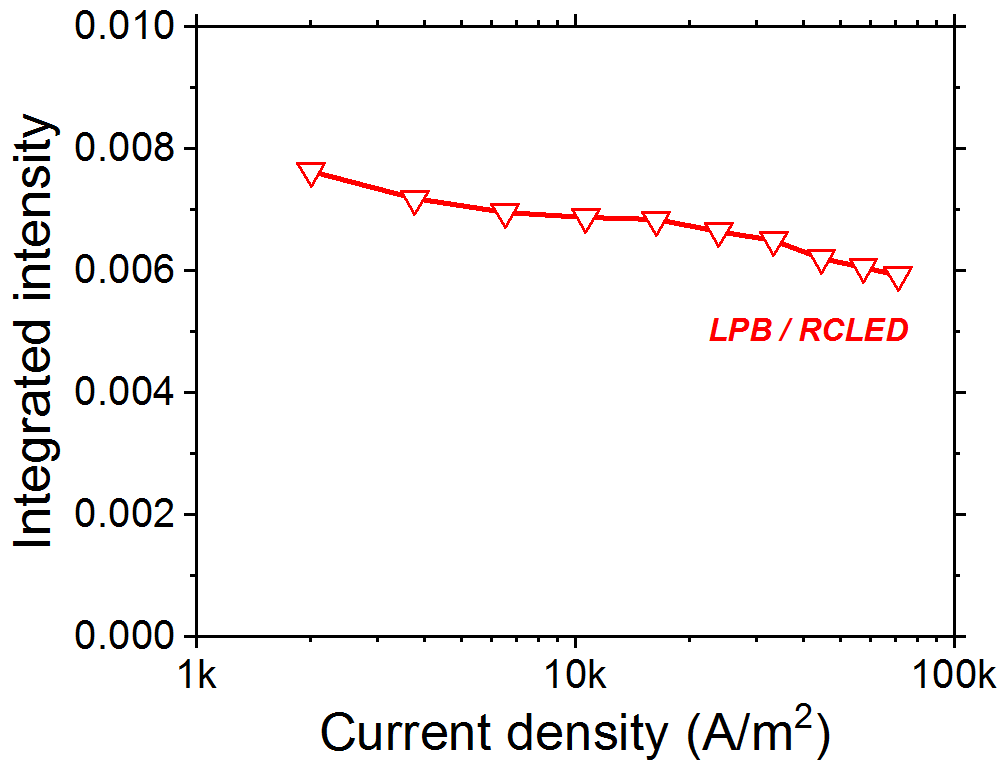
**

Figure S8: LPB/RCLED integrated emission intensity as a function of LED current density

angular range of ±37°. Here, we plot LPB/RCLED emission intensity as a function of LED current density (corresponding to a C.W. drive voltage between 2 – 3 V). It can be seen that the relative intensity of LPB / RCLED emission undergoes a reduction of around 30% over the injection range. We believe that this effect does not result from an intrinsic non-linear process within the device, but most likely originates from thermally assisted photo-oxidation of the Zn-PCN component of the device which was tested in air without encapsulation.
